# Effect of Boiling, Steaming and Microwaving on the Antioxidant and Antibacterial Properties of *Parkia speciosa* Seeds

**DOI:** 10.1007/s11130-025-01351-6

**Published:** 2025-04-16

**Authors:** Lakeisha Fidelia Limas, Ethel Jeyaseela Jeyaraj, Khuen Yen Ng, Wee Sim Choo

**Affiliations:** 1 School of Science, Monash University Malaysia, Jalan Lagoon Selatan, Bandar Sunway, Selangor Malaysia; 2 School of Pharmacy, Monash University Malaysia, Jalan Lagoon Selatan, Bandar Sunway Selangor, Malaysia

**Keywords:** Antioxidant, Antimicrobial, Bitter bean, Cyclic polysulfide, *Parkia speciosa*, Stink bean

## Abstract

This study investigated the effects of boiling, steaming, and microwaving on the total phenolic content (TPC), cyclic polysulfide content, antioxidant, and antibacterial properties of *Parkia speciosa* seeds. Ferric reducing antioxidant power (FRAP) and 1,1-diphenyl-2-picrylhydrazyl (DPPH) free radical scavenging activity of the seeds were evaluated. The main antibacterial components of *P*. *speciosa* seeds, cyclic polysulfides were characterized using Gas Chromatography-Mass Spectrometry analysis and evaluated using a broth microdilution method against seven gram-positive and five gram-negative bacterial strains. Microwaving displayed the highest decrease in TPC, followed by boiling, while steaming retained most TPC. Thermal treatment did not affect DPPH free radical scavenging activity but significantly reduced the FRAP of the seeds. Uncooked seeds exhibited significant antibacterial activity against the 12 bacterial strains with minimum inhibitory concentration (MIC) values of 5–40 mg/mL. Thermal treatment of the seeds displayed reduced antibacterial activity, a decreased amount of 1,2,4,5-tetrathiane whereas 1,3,5-trithiane and 1,2,5,6-tetrathiocane were detected.

## Introduction

*Parkia speciosa*, also known as bitter or stink bean is a leguminous plant belonging to the *Fabaceae* family [1, 2]. *P*. *speciosa* is known as petai in Malaysia and Indonesia whereas it is known as sator and u’pang in Thailand and the Philippines, respectively. *P*. *speciosa* tree can grow up to 40 m in height and 100 cm in diameter. As for the stalks, green, flat, and long pods can grow up to 45 cm long and 6 cm wide. These pods can contain between 10 and 18 light green seeds [1, 3]. *P. speciosa* plant is usually found in lowland tropical rainforests. Still, it can sometimes be found in tall secondary forests or on sandy, loamy, and podzolic soils or freshwater swamp forests, riverbanks and waterlogged places [3].

*P*. *speciosa* seeds can be eaten raw or cooked. The seeds have been used to treat myriad ailments such as headache, hypertension, kidney problems and diabetes [4]. The seeds are believed to possess antioxidant, antitumor, antimutagenicity, antimicrobial and hypoglycemic activities [1, 5–7]. *P*. *speciosa* seeds contain carbohydrates, protein, fat, vitamins, minerals [1, 8, 9], phenolic compounds such as caffeic, ferulic, gallic, *p*-coumaric and trans-cinnamic acid, as well as fatty acids, such as arachidonic, behenic, elaidic, hydnocarpic, lauric, linoleic, myristic, oleic, palmitic, palmitoleic, stearic, stearolic and undecanoic acids [4]. Besides, alkaloids, saponins, and terpenoids such as campesterol, lupeol, β-sitosterol, stigmasterol and squalene were present in the seeds. Cyclic polysulfides, namely hexathionine, pentathiocane, pentathiopane, tetrathiane, tetrathiepane, and trithiolane, were also present in the seeds [10]. These cyclic polysulfides are responsible for the strong, pungent smell of the seeds [4].

Although some vegetables can be eaten raw, most vegetables are routinely cooked before consumption [11]. Common domestic cooking methods include boiling, frying, steaming, and microwaving [12]. Cooking is important to ensure food safety via the destruction of microorganisms and natural toxins like cyanide in vegetables. Cooking improves the food’s palatability and may induce chemical, physical, and biological modifications that cause sensory, textural, and nutritional changes. Cooking can lead to undesirable nutrient losses due to formation of undesirable compounds, chemical reactions, texture loss and discoloration [11]. Cooking may affect the bioavailability of important bioactive compounds in vegetables [12]. For example, denatured proteins are more digestible and readily absorbable by the human gastrointestinal system [11]. Besides, cooking can affect the antioxidant content and antioxidant activity in vegetables [13].

Despite the multiple reports on the antioxidant and antimicrobial properties of *P*. *speciosa* seeds, there are limited studies that investigate the effects of cooking methods on the antioxidant and antibacterial properties of *P*. *speciosa* seeds. This study aims to determine the effect of boiling, steaming and microwaving on the antioxidant and antibacterial properties of *P. speciosa* seeds. Specifically, the total phenolic content, cyclic polysulfide content, free radical scavenging activity, ferric reducing antioxidant power, and minimum inhibitory concentration against 12 strains of bacteria of *P*. *speciosa* seeds were determined.

## Materials and Methods

### Samples

Fresh *P*. *speciosa* pods were purchased from a wet market in Bandar Sunway, Selangor, Malaysia. Seven gram-positive and five gram-negative bacteria (Table 1) were obtained from American Type Culture Collection (ATCC, Manassas, USA).

**Table 1:** Minimum inhibitory concentration (MIC) values (mg/mL) of *Parkia speciosa* seed extracts subjected to different treatments

Bacteria	ATCC no.	MIC value (mg/mL)		
		Uncooked	M5, M10, S5, S10, B5, B10	CAM
*Gram-positive*				
*Staphylococcus aureus*	6538P	20	> 40	0.006
*Staphylococcus aureus*	29,213	20		0.006
MRSA	43,300	10		0.013
*Enterococcus faecalis*	29,212	5		0.006
VRE	700,802	20		0.05
*Bacillus cereus*	14,579	10		0.003
*Bacillus subtilis*	8188	10		0.006
*Gram-negative*				
*Shigella flexneri*	12,022	10		0.003
*Salmonella* Typhimurium	14,028	40		0.003
*Klebsiella pneumoniae*	BAA-1705	40		0.006
*Escherichia coli*	25,922	20		0.002
*Pseudomonas aeruginosa*	BAA-2110	> 40		0.063

MIC values are the means of duplicate. ATTC: American Type Culture Collection; M5: microwaved 5 min; M10: microwaved 10 min; S5: steamed 5 min; S10: steamed 10 min; B5: boiled 5 min; B10: boiled 10 min; MRSA: methicillin-resistant *Staphylococcus aureus*; VRE: vancomycin-resistant Enterococci; CAM: chloramphenicol

### Sample Preparation

*P. speciosa* pods were peeled to obtain the seeds. Boiling, steaming and microwaving of the seeds were performed according to the method of Wachtel-Galor et al. [14] with some modifications. Uncooked seeds were the control. Boiling was performed using 10 g of seeds in 250 mL of distilled water at 100 ℃ in a glass beaker for 5–10 min. Steaming was performed using 10 g of seeds in a food steamer for 5–10 min. Microwaving was performed using 10 g of seeds in 200 mL of distilled water in a glass beaker for 5–10 min using a household microwave (Model M7017P-B, Mitsumaru, Japan) with a rated frequency of 50 Hz, rated voltage of 240 VAC, and rated output of 700 W. After each cooking, the seeds were drained and proceeded to extraction.

### Sample Extraction for Antioxidant Properties Analysis

*P. speciosa* seeds were extracted according to the method of Choo et al. [15] with some modifications. Each sample of *P*. *speciosa* seeds (10 g) with or without cooking was blended with 100 mL of 50% ethanol for 2 min (30 s intermittent stops between 10 s of blending) to reduce heat production using a Waring blender (Model MX110XT11CE, Stamford, USA). The homogenized samples were centrifuged at 5000 × g at 20 ℃ for 25 min. Next, the supernatants were filtered with a Whatman filter paper. The filtered supernatant was then stored at 4 ℃ until further analysis of the total phenolic content and antioxidant activities within the same day. Drying of one portion of the filtered supernatant was performed under reduced pressure using a rotary evaporator at 45 ‘C, followed by freeze-drying for 48 h and kept at -80 °C until broth microdilution assay.

### Total Phenolic Content (TPC)

TPC was determined according to the method of Choo et al. [15]. Samples of 0.3 mL were mixed with 1.2 mL of 7.5% (w/v) sodium carbonate solution and 1.5 mL of Folin-Ciocalteu’s phenol reagent in a test tube. The mixture was left to stand for 30 min in the dark at room temperature before absorbance measurement at 765 nm. TPC was expressed as gallic acid equivalent in mg per g (mg GAE/g) of seeds.

### Free Radical Scavenging Activity

The free radical scavenging activity was conducted according to the method of Vidana Gamage & Choo [16]. DPPH (1,1-diphenyl-2-picrylhydrazyl) solution was prepared with 5.9 mg of DPPH in 100 mL of 100% methanol. Next, 250 µL of the sample was mixed with 1 mL of the DPPH solution and allowed to stand for 30 min in the dark at room temperature. After that, the absorbance of the sample was measured at 517 nm. The free radical scavenging activity was calculated as follows:

$$\eqalign{& {\rm{Percentage}}\,{\rm{of}}\,{\rm{free}}\,{\rm{radical}}\,{\rm{scavenging}}\,{\rm{activity}} \cr & {\rm{ = (Absorbance}}\,{\rm{of}}\,{\rm{control - Absorbance}}\,{\rm{of}}\,{\rm{sample)/}} \cr & \quad {\rm{Absorbance}}\,{\rm{of}}\,{\rm{control \times 100}} \cr} $$

### Ferric Reducing Antioxidant Power (FRAP)

FRAP was determined according to the method of Loh & Lim [17]. A sample of 400 µL was added with 1 mL of 1% (w/v) potassium ferricyanide and 1 mL of 0.2 M pH 6.6 phosphate buffer. The mixture was incubated at 50 ℃ for 20 min followed by the addition of 1 mL trichloroacetic acid (10% w/v). Next, the mixture (1 mL) was mixed with 200 µL of ferric chloride (0.1% w/v) and 1 mL of distilled water, and allowed to stand for 30 min in the dark at room temperature. The absorbance was measured at 700 nm. FRAP was calculated as follows:

$${\rm{FRAP = Absorbance}}\,{\rm{of}}\,{\rm{sample - Absorbance}}\,{\rm{of}}\,{\rm{blank}}$$

### Oil Extraction for Gas Chromatography-Mass Spectrometry (GC-MS) Analysis

The oil extraction was done following the method of Tocmo et al. [18] with some modifications. *P. speciosa* seeds (150 g), with or without cooking, were added with an equal amount of milli-Q water. Homogenization was carried out using a blender (Model MX110XT11CE, Stamford, USA). To minimize heat production, 10 s of blending for a total of 1 min with intermittent stops of 20 s was carried out. This mixture was transferred into a separatory flask and shaken for 5 min. Next, 200 mL of hexane was added and the flask was shaken for another 10 min. After this, the mixture was stored at -80 ℃ overnight to freeze the aqueous portion. The organic layer was collected, subjected to concentration using a rotary evaporator at 40 ℃, and then dried over anhydrous sodium sulfate at room temperature overnight. The oil extract was stored at -20 ℃ until further analysis.

### GC-MS Analysis of *P. speciosa* Seed Oil

GC-MS analysis of *P*. *speciosa* seed oil was performed using an Agilent Technologies 7890 A gas chromatography system attached with an Agilent 5975 C MS unit. An HP-5MS column (Santa Clara, CA, USA) with 30 m × 0.25 mm internal diameter and 0.25 μm film thickness was used. Before injection into the GC system, each oil was diluted with dichloromethane (1:4) and then filtered through a 0.45 μm polytetrafluoroethylene (PTFE) membrane filter. Injection of a sample (1.0 µL) onto the GC system was carried out in a splitless mode using an autosampler Agilent 7683B injector. The injector temperature was set at 250 ℃, and the carrier gas was helium (99.99%) at a flow rate of 1 mL/min. The oven temperature was 50 ℃ for 3 min, ramped up to 250 ℃ at 4 ℃/min, and held at 250 ℃ for 5 min. The ion source temperature was 200 ℃ and the mass range was 40 to 500. The MS transfer line temperature was 270 ℃.

### Minimum Inhibitory Concentration (MIC)

MIC was determined using a broth microdilution method of the Clinical and Laboratory Standards Institute [19] with some modifications. Firstly, samples were filtered with a sterile 0.22 μm PTFE membrane filter and serial doubling dilution was performed before addition to 96 well plates. The volume in each well was 100 µL, with a final working concentration of the samples between 0.3125 mg/mL and 40 mg/mL. Bacteria were grown in 10 mL of BHI broth at 37℃ overnight with shaking at 150 rpm. Standardization using the McFarland standard of 0.08–0.13 absorbance at 625 nm followed by 100 × dilution with BHI broth and loading of the diluted bacterial suspension (100 µL) into each well were carried out. The 96 well plates were incubated for 18–24 h at 37℃. MIC, the lowest concentration of samples required to inhibit the growth of bacteria was observed in a well that appeared to be clear with an unaided eye. The positive control was chloramphenicol.

### Statistical Analysis

All experiments were conducted in independent triplicates. All data, with the exception of MIC data, were analyzed by one-way analysis of variance (ANOVA). A post-hoc Tukey range test was used to determine significance at *p* < 0.05. These analyses were performed using IBM SPSS Statistics 20 (IBM Corporation, Armonk, NY, USA).

## Results and Discussion

### TPC of *P. speciosa* Seed Extract

There were no significant differences using different cooking methods and duration of cooking on the TPC of *P*. *speciosa* seeds (Table 2). However, Wachtel-Galor et al. [14] reported that the duration of cooking can affect the TPC in vegetables, whereby longer cooking time would lead to a greater loss of TPC from the vegetables. When comparing the TPC of cooked samples to that of the uncooked sample, all cooked samples showed a significant reduction in TPC with the exception of steamed samples (Table 2). These results are in accordance with the study conducted by Wachtel-Galor et al. [14] which reported that thermal treatment caused a significant decrease in the TPC of Brassica vegetables (broccoli, choy-sum, and cabbage) with microwave heating showed the strongest effect, whereas steaming caused the least effect. Unlike boiling and microwaving, which expose cooking water directly to the vegetables, steaming utilizes steam from the boiled water to cook the vegetables. Thus, the leaching of phenolic compounds into the cooking water was reduced in steaming.

**Table 2:** Total phenolic content, radical scavenging activity and reducing power of *Parkia speciosa* seed extracts subjected to different treatments

Parkia speciosa	Total Phenolic Content (mg GAE/g)	% Radical Scavenging Activity	Reducing Power
Uncooked	4.54 ± 0.70^b^	85.23 ± 1.77^ab^	0.65 ± 0.11^b^
Microwaved 5 min	1.57 ± 0.38^a^	78.68 ± 4.50^a^	0.32 ± 0.09^a^
Microwaved 10 min	1.72 ± 0.64^a^	86.36 ± 1.63^ab^	0.28 ± 0.07^a^
Steamed 5 min	2.24 ± 1.06^ab^	87.81 ± 0.13^b^	0.37 ± 0.02^a^
Steamed 10 min	2.11 ± 0.65^ab^	86.44 ± 0.27^ab^	0.37 ± 0.03^a^
Boiled 5 min	1.77 ± 0.78^a^	84.42 ± 0.98^ab^	0.28 ± 0.05^a^
Boiled 10 min	1.58 ± 0.34^a^	84.13 ± 1.25^ab^	0.28 ± 0.06^a^

Data are expressed as means of triplicate measurements ± standard deviation. Values with different superscript letters indicate significant differences (*p* < 0.05)

Boiling, microwaving, and steaming were reported to reduce TPC in cabbage, carrot, cauliflower, leek, peas, spinach, and white and yellow turnips [20, 21]. Intriguingly, thermal treatment was reported to increase TPC in vegetables such as broccoli, chili pepper, green beans and pepper [21, 22]. This is most likely due to the release of more phenolic compounds as a result of the thermal breakdown of the cell wall of the vegetables. According to Liu et al. [23] phenolic compounds can be present either in the soluble form or in a complex form with cell wall polysaccharides. Thermal treatment can readily release more phenolic compounds. Although cell wall disruption can release hydrolytic and oxidative enzymes that can alter the structure of molecular compounds in vegetables, this would not be an issue as thermal treatment can deactivate these enzymes and minimize the loss of TPC [24]. The decrease in TPC in vegetables after boiling and microwaving was most likely caused by the leaching of phenolic compounds into the cooking water. The water used for boiling and microwaving was analyzed by Wachtel-Galor et al. [14] and it was reported that more leaching of phenolic compounds from the vegetables into the cooking water occurred with longer cooking time.

### Free Radical Scavenging Activity of *P. speciosa* Seed Extract

There were no significant differences in the free radical scavenging activities in the cooked samples and the uncooked samples of *P*. *speciosa* seeds (Table 2). In addition, a longer cooking duration did not cause lower free radical scavenging activity in the cooked samples. This indicates that phenolic compounds may not be the main compounds that contribute to the free radical scavenging of *P*. *speciosa* seeds, as this activity was not associated with the TPC in cooked samples (Table 2). Free radical scavenging has been reported for peptides in *P. speciosa* seeds [7]. The effect of boiling on the free radical scavenging activity of *P*. *speciosa* seeds is in accordance with the study by Muhialdin et al. [7], whereby there were no significant differences, albeit a longer duration of boiling in the study (45 min) versus this study (5–10 min). However, Turkmen et al. [21] showed that the free radical scavenging activity of broccoli and spinach increased after steaming for 7.5 min and microwaving for 1–1.5 min. The difference is most likely due to different plant sources and the duration of steaming and microwaving.

### Ferric Reducing Antioxidant Power (FRAP) of *P. speciosa* Seed Extract

There were significant reductions in the FRAP after cooking the *P*. *speciosa* seeds (Table 2). These results are in accordance with the study by Wachtel-Galor et al. [14] who reported that the FRAP of cabbage was significantly decreased after boiling, and a study by Ismail et al. [25] who reported a slight decrease of FRAP in cabbage, kale, shallots, and spinach after 1 min of boiling. Among the thermal treatments, the most remarkable FRAP decrease was reported in microwaved vegetables (*i.e.*, broccoli, cabbage, choy sum, and cauliflower) followed by boiled and steamed vegetables [25]. However, a longer cooking duration did not result in lower FRAP in the *P. speciosa* seeds (Table 2). There were also no significant differences in the FRAP of all cooked *P. speciosa* seeds. Several phenolic compounds can react rapidly, like in 4 min, to complete the reduction reaction of the ferric-TPTZ complex, while some other phenolic compounds may take a longer time until 30 min [26]. Pulido et al. [27] found that the absorption of ferric reducing power slowly increased for ferulic acid, ascorbic acid, caffeic acid, tannic acid and quercetin, even after several hours of reaction time. This indicates that a single-point absorption endpoint may not represent a complete reaction [26].

Boiling has been reported to increase FRAP in cooked vegetables including bitter gourd, broccoli, Chinese long bean and water convolvulus [28]. Wachtel-Galor et al. [14] reported more than a double increase in FRAP of steamed vegetables (i.e., broccoli, cauliflower, and choy sum) for 5 min. This could be due to the formation of redox-active secondary plant metabolites from the breakdown of the plant cells. Multiple factors, such as the cooking method, leaching of compounds into the cooking medium, the solvent used for extraction, the degree of heating, and the surface area and pH of the vegetables exposed to oxygen and water, affect FRAP in cooked vegetables [14].

### Cyclic Polysulfides of *P. speciosa* Seed Oil

*Allium* species are known to contain high organosulfide content, predominantly linear organosulfides [18]. In contrast, cyclic polysulfides, rarely occurring in nature, have five to eight-membered rings with three to six sulfur atoms in S-C-S arrangement are present in *P. speciosa* seeds [4, 18]. Cyclic polysulfides contribute to the pungent smell of *P*. *speciosa* seeds and are believed to possess antimicrobial activity [7]. The seven types of cyclic polysulfides detected in *P. speciosa* seeds were hexathiepane, lanthionine, 1,3,5-trithiane, 1,2,4-trithiolane, 1,2,4,5-tetrathiane, 1,2,4,6-tetrathiepane and 1,2,5,6-tetrathiocane (Table 3). Polysulfides with three S-atoms are 1,2,4-trithiolane and 1,3,5-trithiane whereas polysulfides with four S-atoms are 1,2,4,5-tetrathiane, 1,2,4,6-tetrathiepane and 1,2,5,6-tetrathiocane. Polysulfide with five S-atoms is 1,2,3,5,6-pentathiepane or commonly known as lanthionine. Hexathiepane is a polysulfide with six S-atoms.

**Table 3:** Percentage abundance of cyclic polysulfides in *Parkia speciosa* seed oil subjected to different treatments

Cyclic Polysulfides	Mw	Abundance (%)						
		Uncooked	M5	M10	S5	S10	B5	B10
1,2,4-Trithiolane	124	15.53 ± 9.52^a^	16.17 ± 7.15^a^	12.99 ± 17.0^a^	11.02 ± 10.06^a^	20.06 ± 8.16^a^	16.36 ± 3.74^a^	26.24 ± 6.02^a^
1,3,5-Trithiane	138	n.d.	1.97 ± 2.79^a^	2.46 ± 3.47^a^	1.62 ± 2.29^a^	1.85 ± 2.62^a^	2.12 ± 2.99^a^	3.4 ± 4.81^a^
1,2,4,5-Tetrathiane	156	48.23 ± 1.26^b^	17.48 ± 0.11^a^	15.14 ± 0.18^a^	15.56 ± 13.29^a^	19.26 ± 2.06^a^	21.33 ± 2.06^a^	16.77 ± 1.57^a^
1,2,4,6-Tetrathiepane	170	0.27 ± 0.06^a^	2.93 ± 3.8^a^	2.01 ± 2.32^a^	0.82 ± 1.04^a^	1.46 ± 1.58^a^	1.78 ± 2.09^a^	1.81 ± 1.64^a^
1,2,5,6-Tetrathiocane	184	n.d.	0.12 ± 0.17^a^	0.17 ± 0.23^a^	0.62 ± 0.88^a^	0.06 ± 0.08^a^	0.07 ± 0.09^a^	0.13 ± 0.18^a^
Lenthionine	188	20.7 ± 1.39^a^	38.47 ± 5.99^a^	37.0 ± 10.3^a^	26.43 ± 8.6^a^	33.1 ± 8.25^a^	37.44 ± 6.02^a^	30.29 ± 11.28^a^
Hexathiepane	206	0.43 ± 0.61^a^	0.37 ± 0.52^a^	0.3 ± 0.42^a^	0.63 ± 0.5^a^	0.87 ± 0.13^a^	0.67 ± 0.16^a^	0.6 ± 0.05^a^

Data are expressed as mean ± standard deviation. Values with different superscript letters within a row indicate a significant difference (*p* < 0.05). Mw: molecular weight; n.d.: not detected; M5: Microwaved 5 min; M10: Microwaved 10 min; S5: Steamed 5 min; S10: Steamed 10 min; B5: Boiled 5 min; B10: Boiled 10 min

The Maillard reaction is the main route to sulfur compound formation during thermal treatment. L-cysteine is an important precursor to form sulfur compounds [29]. In this study, 1,2,5,6-tetrathiocane was only found in cooked *P. speciosa* seeds (Table 3). According to Yu et al. [29], the formation of 1,2,5,6-tetrathiocane occurs as a result of thermal degradation of L-cysteine at 140℃ and pH 8. There were no significant differences in hexathiepane, lenthionine, 1,2,4-trithiolane and 1,2,4,6-tetrathiepane in uncooked and cooked *P. speciosa* seeds (Table 3). These results suggest that these four polysulfides were thermally stable. However, the abundance of 1,2,4,5-tetrathiane decreased significantly due to cooking (Table 3).

There were seven polysulfides detected in cooked *P. speciosa* seeds, but only five polysulfides were detected in the uncooked *P. speciosa* seeds, with 1,3,5-trithiane and 1,2,5,6-tetrathionate being not detected (Table 3), Miyazawa & Osman [30] reported that 1,2,4-trithiolane and 1,2,4,5-tetrathiane were detected as the major polysulfide in *P. speciosa* seeds. On the other hand, Azizi et al. [31] reported lenthionine and 1,2,4,5-tetrathiane were detected in *P. speciosa* seeds. Only 1,2,4,5-tetrathiane from the uncooked seeds was significantly higher (*p* < 0.05) than those from the cooked *P. speciosa* seeds (Table 3).

Among the five polysulfides present in the uncooked seeds, 1,2,4,5-tetrathiane was found to be most abundant, followed by lenthionine, 1,2,4-trithiolane, hexathipane, and 1,2,4,6-tetrathiepane (Table 3). Tocmo et al. [18] also reported the presence of similar polysulfides with 1,2,4-tritholane as the most abundant polysulfide in uncooked *P. speciosa* seeds. However, in this study, 1,2,3,5-tetrathiane and dimethyl tetrasulfide (acyclic polysulfides) were not detected. This may be due to different extraction methods. According to Tocmo et al. [18] the types of organosulfides extracted using hydrodistillation of *P*. *speciosa* oil were different from those using solvent extraction. The variations in the types of organosulfides and differences in the concentration of each organosulfide were also reported in other studies [30, 31].

### MIC of *P. speciosa* Seed Extract

The most susceptible bacteria among the 12 bacterial strains to uncooked *P. speciosa* seed extracts was the gram-positive *Enterococcus faecalis* as the MIC value was the lowest (Table 1). The least susceptible bacteria among the 12 bacterial strains to uncooked *P. speciosa* seed extracts was the gram-negative *Pseudomonas aeruginosa* (Table 1). This may be due to the cell walls of gram-negative bacteria being surrounded by an outer lipopolysaccharide membrane that can restrict the diffusion of hydrophobic compounds through it [10]. Therefore, gram-positive bacteria have lower outer layer impermeability compared to gram-negative bacteria. Ghasemzadeh et al. [10] reported lower MIC values for *Staphyloccocus aureus* (0.04–0.08 mg/mL), *Esherichia coli* were > 0.1 mg/mL and *Salmonella Typhimurium* ranged from 0.08 to > 0.1 mg/mL. This could be due to the different methods of extraction of *P. speciosa* seeds. This study extracted the *P. speciosa* seeds without drying whereas the *P. speciosa* seeds used by Ghasemzadeh et al. [10] were dried at 45℃ and ground into powder before extraction. This may affect the composition of the extracted compounds, resulting in different MIC values.

*P. speciosa* seed extracts that were subjected to different cooking methods showed weaker antibacterial activity than uncooked seed extracts (Table 1). The MIC values of *P. speciosa* seed extracts against the 12 tested bacterial strains were more than 40 mg/mL for each cooking method. The weaker antibacterial activity exhibited by cooked seed extracts is most likely due to the significant decrease in 1,2,4,5-tetrathiane (Table 3). Besides, boiling and microwaving decreased the TPC of *P. speciosa* seeds (Table 2). Phenolic acid such as gallic acid, and flavonols such as quercetin and kaempferol in *P. speciosa* seeds, are the compounds that inhibit gram-positive and gram-negative bacteria growth [10]. These phenolic compounds might be degraded by the different cooking methods.

Hexathionine and trithiolane are two cyclic polysulfides that are known to contribute to the antibacterial properties of *P*. *speciosa* seeds [32]. In this study, hexathionine compounds were not detected but trithiolane (1,2,4-trithiolane) was found in uncooked *P*. *speciosa* seeds (Table 3). In uncooked *P*. *speciosa* seeds, 1,2,4-trithiolane (15.53%) was found to be the third most abundant cyclic polysulfide after 1,2,4,5-tetrathiane (48.23%) and lenthionine (20.70%). These cyclic polysulfide compounds likely contribute to the antibacterial activity of the seeds, but this requires further investigation.

## Conclusions

Microwaved and boiled *P*. *speciosa* seeds showed a significant reduction in TPC whereas steaming was able to retain most of the TPC in *P*. *speciosa* seeds compared to uncooked samples. There were no significant differences in the free radical scavenging activity of uncooked and cooked samples. However, there was a significant reduction in the reducing power of *P*. *speciosa* seeds due to microwaving, steaming and boiling, but there were no significant differences in using these cooking methods. There were no significant effects on the abundance of cyclic polysulfides due to microwaving, steaming and boiling of *P*. *speciosa* seeds except for a significant reduction in 1,2,4,5-tetrathiane. As a result of using microwaving, steaming and boiling, compounds such as 1,3,5-trithiane and 1,2,5,6-tetrathiocane were detected compared to uncooked samples. The cooked *P*. *speciosa* seeds exhibited weaker antibacterial properties than the uncooked seeds. This study demonstrated that steaming is the best cooking method to retain the beneficial phenolic compounds. Since cooking alters the chemical composition and reduces antioxidant and antibacterial properties, different preparation methods may need to be considered depending on whether *P. speciosa* seeds are consumed for their taste, health benefits, or medicinal potential. Consuming uncooked seeds may be the best way to obtain their beneficial properties. Other preparation methods that can be investigated include roasting, frying, pickling, or fermentation.

## Data Availability

The datasets generated during and/or analyzed during the current study are included in this article.

## References

[1] Kamisah Y, Othman F, Qodriyah HMS, Jaarin K (2013) *Parkia speciosa* Hassk: A potential phytomedicine. Evid Based Complement Alternat Med 2013:709028. 10.1155/2013/709028 23956777 10.1155/2013/709028 PMC3730359

[2] Azemi AK, Nordin ML, Hambali KA, Noralidin NA, Mokhtar SS, Rasool AHG (2022) Phytochemical contents and Pharmacological potential of *Parkia speciosa* Hassk. For diabetic vasculopathy: A review. Antioxidants 11:431 35204313 10.3390/antiox11020431 PMC8869085

[3] Orwa C, Mutua A, Kindt R, Jamnadass R, Anthony S (2009) Agroforestree Database: A tree reference and selection guide. version 4.0. Retrieved from https://apps.worldagroforestry.org/treedb/AFTPDFS/Parkia_speciosa.PDF. Accessed April 19, 2024

[4] Singhania N, Chhikara N, Bishnoi S, Garg MK, Panghal A (2020) Bioactive compounds of Petai beans (*Parkia speciosa* Hassk). In: Murthy HN, Paek KY (eds) Bioactive compounds in underutilized vegetables and legumes. Springer Cham, Switzerland

[5] Jamaluddin F, Mohamed S, Lajis MN (1994) Hypoglycaemic effect of *Parkia speciosa* seeds due to the synergistic action of b-sitosterol and stigmasterol. Food Chem 49:339e345

[6] Ko HJ, Ang LH, Ng LT (2014) Antioxidant activities and polyphenolic constituents of bitter bean Parkia speciosa. Int J Food Prop 17:1977–1986

[7] Muhialdin BJ, Rani NFA, Hussin ASM (2020) Identification of antioxidant and antibacterial activities for the bioactive peptides generated from bitter beans (*Parkia speciosa*) via boiling and fermentation processes. LWT 131:109776

[8] Chen AY, Chen YC (2013) A review of the dietary flavonoid, Kaempferol on human health and cancer chemoprevention. Food Chem 138:2099–2107 23497863 10.1016/j.foodchem.2012.11.139 PMC3601579

[9] Wootton-Beard PC, Moran A, Ryan L (2011) Stability of the total antioxidant capacity and total polyphenol content of 23 commercially available vegetable juices before and after *in vitro* digestion measured by FRAP, DPPH, ABTS and Folin-Ciocalteu methods. Food Res Int 44:217–224

[10] Ghasemzadeh A, Jaafar HZ, Bukhori MFM, Rahmat MH, Rahmat A (2018) Assessment and comparison of phytochemical constituents and biological activities of bitter bean (*Parkia speciosa* Hassk.) collected from different locations in Malaysia. Chem Cent J 12:1–9 29417254 10.1186/s13065-018-0377-6 PMC5803156

[11] Palermo M, Pellegrini N, Fogliano V (2014) The effect of cooking on the phytochemical content of vegetables. J Sci Food Agric 94:1057–1070 24227349 10.1002/jsfa.6478

[12] Mehmood A, Zeb A (2020) Effects of different cooking techniques on bioactive contents of leafy vegetables. Int J Gastron Food Sci 22:100246

[13] Mulțescu M, Zachia M, Belc N, Burnichi F, Israel-Roming F (2020) Antioxidants in fresh and cooked broccoli (*Brassica Oleracea* Var. Avenger) and cauliflower (*Brassica Oleracea* Var. Alphina F1). Sci Bull Ser F Biotechnol 24:107–113

[14] Wachtel-Galor S, Wong KW, Benzie IF (2008) The effect of cooking on *Brassica* vegetables. Food Chem 110:706–710

[15] Choo WS, Yap JY, Chan SY (2014) Antioxidant properties of two varieties of bitter gourd (*Momordica charantia*) and the effect of blanching and boiling on them. Pertanika J Trop Agric Sci 37:121–131

[16] Vidana Gamage GC, Choo WS (2023) Thermal and pH stability of natural anthocyanin colourant preparations from black Goji berry. Food Chem Adv 2:100236

[17] Loh ZH, Lim YY (2018) Drying effects on antioxidant activity, enzyme activity, and phytochemicals of avocado (*Persea americana*) leaves. J Food Process Preserv 42, e13667

[18] Tocmo R, Liang D, Wang C, Poh J, Huang D (2016) Organosulfide profile and hydrogen sulfide-releasing capacity of stinky bean (*Parkia speciosa*) oil: effects of pH and extraction methods. Food Chem 190:1123–1129 26213085 10.1016/j.foodchem.2015.06.072

[19] Clinical and Laboratory Standards Institute (CLSI) (2018) Methods for dilution antimicrobial susceptibility tests for bacteria that grow aerobically. 11th ed. CLSI Standard M07, Wayne, USA

[20] Rani EP, Fernando RRS (2016) Effect of cooking on total antioxidant activity in selected vegetables. Int J Home Sci 2:218–222

[21] Turkmen N, Sari F, Velioglu YS (2005) The effect of cooking methods on total phenolics and antioxidant activity of selected green vegetables. Food Chem 93:713–718

[22] Shaimaa GA, Mahmoud MS, Mohamed MR, Emam AA (2016) Effect of heat treatment on phenolic and flavonoid compounds and antioxidant activities of some Egyptian sweet and Chilli pepper. Nat Prod Chem Res 4:1000218

[23] Liu X, Le Bourvellec C, Renard CM (2020) Interactions between cell wall polysaccharides and polyphenols: effect of molecular internal structure. Compr Rev Food Sci Food Saf 19:3574–3617 33337054 10.1111/1541-4337.12632

[24] Dewanto V, Wu X, Adom KK, Liu RH (2002) Thermal processing enhances the nutritional value of tomatoes by increasing total antioxidant activity. J Agric Food Chem 50:3010–3014 11982434 10.1021/jf0115589

[25] Ismail A, Marjan ZM, Foong CW (2004) Total antioxidant activity and phenolic content in selected vegetables. Food Chem 87:581–586

[26] Prior RL, Wu X, Schaich K (2005) Standardized methods for the determination of antioxidant capacity and phenolics in foods and dietary supplements. J Agric Food Chem 53:4290–4302 15884874 10.1021/jf0502698

[27] Pulido R, Bravo L, Saura-Calixto F (2000) Antioxidant activity of dietary polyphenols as determined by a modified ferric reducing/antioxidant power assay. J Agric Food Chem 48:3396–3402 10956123 10.1021/jf9913458

[28] Ng ZX, Chai JW, Kuppusamy UR (2011) Customized cooking method improves total antioxidant activity in selected vegetables. Int J Food Sci Nutr 62:158–163 21250903 10.3109/09637486.2010.526931

[29] Yu AN, Tan ZW, Wang FS (2012) Mechanism of formation of sulphur aroma compounds from L-ascorbic acid and L-cysteine during the Maillard reaction. Food Chem 132:1316–1323 29243617 10.1016/j.foodchem.2011.11.111

[30] Miyazawa M, Osman F (2001) Headspace constituents of *Parkia speciosa* seeds. Nat Prod Lett 15:171–176 11858549 10.1080/10575630108041277

[31] Azizi CM, Salman Z, Norulain NN, Omar AM (2008) Extraction and identification of compounds from *Parkia speciosa* seeds by supercritical carbon dioxide. J Chem Nat Res Eng 2:153–163

[32] Zaini NA, Mustaffa F (2017) *Parkia speciosa* as valuable, miracle of nature. Asian J Med Health 2:1–9

